# Coarse-graining molecular dynamics: stochastic models with non-Gaussian force distributions

**DOI:** 10.1007/s00285-019-01433-5

**Published:** 2019-09-21

**Authors:** Radek Erban

**Affiliations:** Mathematical Institute, Radcliffe Observatory Quarter, University of Oxford, Woodstock Road, Oxford, OX2 6GG UK

**Keywords:** Multiscale modelling, Coarse-graining, Molecular dynamics, Brownian dynamics, 82C31, 92C40, 60H10, 65C35, 60G15

## Abstract

Incorporating atomistic and molecular information into models of cellular behaviour is challenging because of a vast separation of spatial and temporal scales between processes happening at the atomic and cellular levels. Multiscale or multi-resolution methodologies address this difficulty by using molecular dynamics (MD) and coarse-grained models in different parts of the cell. Their applicability depends on the accuracy and properties of the coarse-grained model which approximates the detailed MD description. A family of stochastic coarse-grained (SCG) models, written as relatively low-dimensional systems of nonlinear stochastic differential equations, is presented. The nonlinear SCG model incorporates the non-Gaussian force distribution which is observed in MD simulations and which cannot be described by linear models. It is shown that the nonlinearities can be chosen in such a way that they do not complicate parametrization of the SCG description by detailed MD simulations. The solution of the SCG model is found in terms of gamma functions.

## Introduction

With increased experimental information on atomic or near-atomic structure of biomolecules and intracellular components, there has been a growing need to incorporate such microscopic data (coming from X-ray crystallography, NMR spectroscopy or cryo-electron microscopy) into dynamical models of intracellular processes. A common approach is to use molecular dynamics (MD) simulations based on classical molecular mechanics. Such MD models are written as relatively large systems of ordinary or stochastic differential equations for the positions and velocities of individual atoms, which can also be subject to algebraic constraints (Leimkuhler and Matthews 2015; Lewars 2016). Although all-atom MD simulations of systems consisting of a million of atoms have been reported in the literature (Tarasova et al. 2017; Farafonov and Nerukh 2019), such simulations are restricted to relatively small computational domains, which are up to tens of nanometres long. It is beyond the reach of state-of-the-art computers to simulate intracellular processes which include transport of molecules over micrometers, because this would require simulations of trillions of atoms (Erban and Chapman 2019).

An example is modelling of calcium ($\hbox {Ca}^{2+}$) dynamics. On one hand, at the macroscopic level, $\hbox {Ca}^{2+}$ waves can propagate between cells over hundreds of micrometres and Kang and Othmer (2009) developed a model of $\hbox {Ca}^{2+}$ waves in a network of astrocytes. It builds on previous modelling work by Kang and Othmer (2007) describing intracellular $\hbox {Ca}^{2+}$ dynamics as a system of differential equations for concentrations of chemical species involved, including inositol 1,4,5-trisphosphate ($\hbox {IP}_3$), a chemical signal that binds to the $\hbox {IP}_3$ receptor to release $\hbox {Ca}^{2+}$ ions from the endoplasmic reticulum. On the other hand, at the atomic level, Hamada et al. (2017) recently solved $\hbox {IP}_3$-bound and unbound structures of large cytosolic domains of the $\hbox {IP}_3$ receptor by X-ray crystallography and clarified the $\hbox {IP}_3$-dependent gating mechanism through a unique leaflet structure.

Although it is not possible to incorporate such a detailed information into $\hbox {Ca}^{2+}$ modelling by using all-atom MD in the entire intracellular space, there is still potential to design multiscale (multi-resolution) models which compute $\hbox {Ca}^{2+}$ dynamics with the resolution of individual $\hbox {Ca}^{2+}$ ions. Dobramysl et al. (2016) implement such a methodology at the Brownian dynamics (BD) level to study $\hbox {Ca}^{2+}$ puff statistics stemming from $\hbox {IP}_3$ receptor channels. Denoting the position of an individual $\hbox {Ca}^{2+}$ ion by ${\mathbf {X}} \equiv (X_1,X_2,X_3)$, its diffusive BD trajectory is given by

1 $$\begin{aligned} \mathrm{d}X_i = \sqrt{2 D} \; \mathrm{d}W_i, \qquad \text{ for } \;\; i=1,2,3, \end{aligned}$$

where *D* is the diffusion constant and $$W_i,$$$$i=1,2,3,$$ are three independent Wiener processes. Since individual positions of $$\hbox {Ca}^{2+}$$ ions are only needed in the vicinity of channel sites, Dobramysl et al. (2016) model diffusion of ions far away of the channel by a coarser model, utilizing the two-regime method developed by Flegg et al. (2012). This method enables efficient simulations with the BD level of resolution by coarse-graining the BD model in those parts of the simulation domain, where the coarse-grained model can be safely used without introducing significant numerical errors (Flegg et al. 2014, 2015; Robinson et al. 2015).

Although BD models or their multi-resolution extensions simulate individual molecules of chemical species involved, the binding of $\hbox {Ca}^{2+}$ ions to channel sites or other interactions between molecules are only described using relatively coarse probabilistic approaches. For example, the BD model of Dobramysl et al. (2016) describes interactions in terms of reaction radii and binding probabilities as implemented by Erban and Chapman (2009) and Lipková et al. (2011). Atomic-level information is not included in BD models. In order to use this information, multi-resolution methodologies have to consider MD simulations in parts of the simulation domain. In the case of ions, such a multi-resolution scheme has been developed by Erban (2016), where an all-atom MD model of ions in water is coupled with a stochastic coarse-grained (SCG) description of ions in the rest of the computational domain.

The accuracy and efficiency of such multi-resolution methodologies depend on the quality of the SCG description of the underlying MD model. In this paper, we present and analyze a class of SCG models which can be used to fit non-Gaussian distributions estimated from all-atom MD simulations. While the velocity distribution of the coarse-grained particle can be well approximated by a Gaussian (normal) distribution in our MD simulations, this is not the case of the force distribution. Non-Gaussian force distributions have also been reported by Shin et al. (2010) and Carof et al. (2014) in their MD simulations of particles in Lennard-Jones fluids. Thus our SCG model is formulated in a way which incorporates a Gaussian distribution for the velocity and a non-Gaussian distribution for the force (acceleration).

Given an integer $N \ge 1$, a coarse-grained particle (for example, an ion) will be described by $(2N+2)$ three-dimensional variables: its position ${\mathbf {X}}$, velocity ${\mathbf {V}}$ and 2*N* auxiliary variables ${\mathbf {U}}_j$ and ${\mathbf {Z}}_j$, where $j=1,2,\ldots ,N$. Denoting ${\mathbf {X}} \equiv (X_1,X_2,X_3)$, ${\mathbf {V}} \equiv (V_1,V_2,V_3)$, ${\mathbf {U}}_j \equiv (U_{j,1},U_{j,2},U_{j,3})$ and ${\mathbf {Z}}_j \equiv (Z_{j,1},Z_{j,2},Z_{j,3})$, the time evolution of the SCG model is given by

2 $$\begin{aligned} \mathrm{d}X_i= & {} V_{i} \, \mathrm{d}t, \qquad \text{ for } \;\; i=1,2,3, \end{aligned}$$

3 $$\begin{aligned} \mathrm{d}V_i= & {} \sum _{j=1}^N U_{j,i} \, \mathrm{d}t, \end{aligned}$$

4 $$\begin{aligned} \mathrm{d}U_{j,i}= & {} \left( -\eta _{j,1} V_i + h_j(Z_{j,i}) \right) \, g_j^\prime (g_j^{-1}(U_{j,i})) \, \mathrm{d}t, \qquad \text{ for } \;\; j=1,2,\ldots ,N, \qquad \end{aligned}$$

5 $$\begin{aligned} \mathrm{d}Z_{j,i}= & {} - \left( \eta _{j,2} \, h_j(Z_{j,i}) + \eta _{j,3} U_{j,i}\right) \, \mathrm{d}t + \eta _{j,4} \; \mathrm{d}W_{j,i}, \end{aligned}$$

where $g_j: {\mathbb {R}} \rightarrow {\mathbb {R}}$ is an increasing differentiable function, $g_j^\prime$ is its derivative, $g_j^{-1}$ is its inverse, $h_j: {\mathbb {R}} \rightarrow {\mathbb {R}}$ is a continuous function and $\eta _{j,k}$ are positive constants for $j=1,2,\ldots ,N$ and $k=1$, 2, 3, 4. We note that some of our assumptions on $g_j$ can be relaxed as long as $g_j^\prime (g_j^{-1}(U_{j,i}))$ appearing in Eq. (4) can be suitably defined.

The SCG description (2)–(5) includes 2*N* functions $g_j$ and $h_j$ and 4*N* additional parameters $\eta _{j,k}$, which can be all adjusted to fit properties of the detailed all-atom MD model. In particular the SCG model (2)–(5) can better match the MD trajectories of ions than the BD description given by Eq. (1), which only has one parameter, diffusion constant *D*, to fit to the MD results.

One of the shortcomings of Eq. (1) is that its derivation from the underlying MD model requires us to consider the limit of sufficiently large times. In particular, we need to discretize Eq. (1) with a relatively large time step, say a nanosecond, to use it as a description of the trajectory of an ion. Since the typical time step of an all-atom MD model is a femtosecond, it is difficult to design a multi-resolution scheme which would replace all-atom MD simulations by Eq. (1) in parts of the computational domain. The SCG model (2)–(5) can be used to fit not only the diffusion constant *D* but other important properties of all-atom MD models, which improves the accuracy of the SCG model at time steps comparable with the MD timestep.

SCG models can be constructed using a relatively automated procedure by postulating that an ion interacts with additional ‘fictitious particles’. Such a methodology has been applied to coarse-grained modelling of biomolecules by Davtyan et al. (2015, 2016) to improve the fit between an MD model and the dynamics on a coarse-grained potential surface. They use fictitious particles with harmonic interactions with coarse-grained degrees of freedom (i.e. they add quadratic terms to the potential function of the system and linear terms to equations of motions) and each fictitious particle is also subject to a friction force and noise. An application of such an approach to ions leads to systems of linear stochastic differential equations (SDEs) and can be used, after some transformation, to obtain a simplified version of the SCG model (2)–(5), where functions $g_j$ and $h_j$ are given as identities, i.e. $g_j(y) = h_j(y)=y$ for $y \in {\mathbb {R}}$ and $j=1,2,\ldots ,N$. Using this simplifying assumption in the SCG model (2)–(5), we obtain

6 $$\begin{aligned} \mathrm{d}X_i= & {} V_{i} \, \mathrm{d}t, \qquad \text{ for } \;\; i=1,2,3, \end{aligned}$$

7 $$\begin{aligned} \mathrm{d}V_i= & {} \sum _{j=1}^N U_{j,i} \, \mathrm{d}t, \end{aligned}$$

8 $$\begin{aligned} \mathrm{d}U_{j,i}= & {} \left( -\eta _{j,1} V_i + Z_{j,i} \right) \, \mathrm{d}t, \qquad \text{ for } \;\; j=1,2,\ldots ,N, \qquad \end{aligned}$$

9 $$\begin{aligned} \mathrm{d}Z_{j,i}= & {} - \left( \eta _{j,2} Z_{j,i} + \eta _{j,3} U_{j,i} \right) \, \mathrm{d}t + \eta _{j,4} \; \mathrm{d}W_{j,i} . \end{aligned}$$

This is a linear system of SDEs with 4*N* parameters. It has been shown by Erban (2016) that such models can fit an increasing number of properties of all-atom MD simulations as we increase *N*. For example, the linear SCG model (6)–(9) can be used to fit the diffusion constant *D* and second moments of the velocity and the force for $N=1$, while the velocity autocorrelation function can better be fitted for larger values of *N*, e.g. for $N=3$. However, there are other properties of MD simulations which cannot be captured by linear models even if consider arbitrarily large *N*. They include, for example, all distributions which are not Gaussian. This motivates the introduction of general functions $h_j$ and $g_j$ in the SCG model (2)–(5).

Considering the SCG model (2)–(5) in its full generality, it can capture more interesting dynamics. However, coarse-grained models can only be useful if they can be easily parametrized. Thus in our analysis, we focus on choices of functions $g_j$ and $h_j$ which both improve the properties of the SCG description and do not complicate its analysis and parametrization. The rest of the paper is organized as follows. In Sect. 2, we consider the linear SCG model (6)–(9) for $N=1$, which is followed in Sect. 3 with the analysis of the linear model for general values of *N*. To get some further insights into the properties of this model, we study its connections with the corresponding generalized Langevin equation. In Sect. 4, we consider the nonlinear SCG model (2)–(5) for $N=1$. We consider specific choices of nonlinearity $g_1$, for which the model can be solved in terms of incomplete gamma functions. This helps us to design three approaches to parametrize the nonlinear SCG model, which are applied to data obtained from MD simulations. We conclude with the analysis of the nonlinear SCG model (2)–(5) for general values of *N* in Sect. 5.

## Linear model for $N=1$ and the generalized Langevin equation

We begin by considering the linear SCG model (6)–(9) for $$N=1$$. To simplify our notation in this section, we will drop some subscripts and denote $$X = X_i,$$$$V = V_i$$, $$U = U_{1,i}$$, $$Z=Z_{1,i}$$, $$W=W_{1,i}$$ and $$\eta _{k} = \eta _{1,k}$$ for $$k=1$$, 2, 3, 4. Then Eqs. (6)–(9) read as follows

10 $$\begin{aligned} \mathrm{d}X= & {} V \, \mathrm{d}t, \end{aligned}$$

11 $$\begin{aligned} \mathrm{d}V= & {} U \, \mathrm{d}t,\end{aligned}$$

12 $$\begin{aligned} \mathrm{d}U= & {} \left( -\eta _{1} V + Z \right) \, \mathrm{d}t, \end{aligned}$$

13 $$\begin{aligned} \mathrm{d}Z= & {} - \left( \eta _{2} Z + \eta _{3} U \right) \, \mathrm{d}t + \eta _{4} \; \mathrm{d}W, \qquad \end{aligned}$$

where *X* is (one coordinate of) the position of the coarse-grained particle *V* is its velocity, *U* is its acceleration, *Z* is an auxiliary variable, $$\mathrm{d}W$$ is white noise and $$\eta _j$$, $$j=1$$, 2, 3, 4, are positive parameters. In order to find the values of four parameters $$\eta _j$$ suitable for modelling ions, Erban (2016) estimates the diffusion constants *D* and three second moments $$\langle V^2 \rangle $$, $$\langle U^2 \rangle $$ and $$\langle Z^2 \rangle $$ from all-atom MD simulations of ions ($$\hbox {K}^+,$$$$\hbox {Na}^+,$$$$\hbox {Ca}^{2+}$$ and $$\hbox {Cl}^-$$) in aqueous solutions. The four parameters of the SCG model (10)–(13) can then be chosen as

14 $$\begin{aligned} \eta _1 = \frac{\langle U^2 \rangle }{\langle V^2 \rangle }, \;\; \eta _2 = \frac{\langle Z^2 \rangle }{D} \left( \frac{\langle V^2 \rangle }{\langle U^2 \rangle } \right) ^{2}, \;\; \eta _3 = \frac{\langle Z^2 \rangle }{\langle U^2 \rangle }, \;\; \eta _4 = \sqrt{\frac{2}{D}} \frac{\langle V^2 \rangle \langle Z^2 \rangle }{\langle U^2 \rangle }. \end{aligned}$$

Then the SCG model (10)–(13) gives the same values of *D*, $\langle V^2 \rangle$, $\langle U^2 \rangle$ and $\langle Z^2 \rangle$ as obtained in all-atom MD simulations.

Since the model (10)–(13) only has four parameters, we can only hope to get the exact match of four quantities estimated from all-atom MD. To get some insights into what we are missing, we will derive the corresponding generalized Langevin equation and study its consequences. The generalized Langevin equation can be written in the form

15 $$\begin{aligned} \frac{\mathrm{d}V}{\mathrm{d}t} = - \int _0^t K(\tau ) \, V(t-\tau ) \, \mathrm{d}\tau + R(t), \end{aligned}$$

where $K: [0,\infty ) \rightarrow {\mathbb {R}}$ is a memory kernel and random term *R*(*t*) satisfies the generalized fluctuation-dissipation theorem, given below in Eq. (21). To derive the generalized Langevin equation (15), consider the two-variable subsystem (12)–(13) of the SCG model. Denoting ${\mathbf {y}} = (U,Z)^\mathrm{T},$ where ${\mathrm {T}}$ stands for transpose, Eqs. (12)–(13) can be written in vector notation as follows

16 $$\begin{aligned} \mathrm{d} {\mathbf {y}} = B \, {\mathbf {y}} \, \mathrm{d}t + \mathbf {b}_{\mathbf {1}} V \, \mathrm{d}t + \mathbf {b}_{\mathbf {2}} \, \mathrm{d}W, \end{aligned}$$

where matrix $B \in {\mathbb {R}}^{2 \times 2}$ and vectors ${\mathbf {b}}_j \in {\mathbb {R}}^{2}$, $j=1,2,$ are given as

$$\begin{aligned} B = \left( \begin{matrix} 0 &{}\quad 1 \\ - \eta _3 &{}\quad -\eta _2 \end{matrix} \right) , \qquad {\mathbf {b}}_1 = \left( \begin{matrix} - \eta _1 \\ 0 \end{matrix} \right) \qquad \text{ and } \qquad {\mathbf {b}}_2 = \left( \begin{matrix} 0 \\ \eta _4 \end{matrix} \right) . \end{aligned}$$

Let us denote the eigenvalues and eigenvectors of *B* as $$\lambda _j$$ and $${\pmb \nu }_j = (1, \lambda _j)^\mathrm{T},$$$$j=1,2,$$ respectively. The eigenvalues of *B* are the solutions of the characteristic polynomial $$ \lambda ^2 + \eta _2 \, \lambda + \eta _3 = 0. $$ They are given by

17 $$\begin{aligned} \lambda _{1} = - \frac{\eta _2}{2} + \mu \quad \; \text{ and } \quad \; \lambda _{2} = - \frac{\eta _2}{2} - \mu \quad \; \text{ where } \quad \; \mu = \sqrt{\frac{\eta _2^2}{4} - \eta _3}. \end{aligned}$$

Since $\eta _2$ and $\eta _3$ are positive parameters,  we conclude that real parts of both eigenvalues are negative. In what follows, we will assume $\eta _2^2 \ne 4 \eta _3$. Then we have two distinct eigenvalues and the general solution of the SDE system (16) can be written as follows

18 $$\begin{aligned} {\mathbf {y}}(t) = \varPhi (t) \, {\mathbf {c}} + \varPhi (t) \int _0^t \varPhi ^{-1}(s) \, {\mathbf {b}_1} V(s) \, \mathrm{d}s + \varPhi (t) \int _0^t \varPhi ^{-1}(s) \, {\mathbf {b}_2} \, \mathrm{d}W, \end{aligned}$$

where ${\mathbf {c}} \in {\mathbb {R}}^{2}$ is a constant vector determined by initial conditions and matrix $\varPhi (t) \in {\mathbb {R}}^{2 \times 2}$ is given as

$$\begin{aligned} \varPhi (t) = (\exp (\lambda _1 t) {\pmb \nu }_1 \; | \; \exp (\lambda _2 t) {\pmb \nu }_2 ) = \left( \begin{matrix} \exp (\lambda _1 t) &{}\quad \exp (\lambda _2 t) \\ \lambda _1 \exp (\lambda _1 t) &{}\quad \lambda _2 \exp (\lambda _2 t) \end{matrix} \right) , \end{aligned}$$

i.e. each column is a solution of the ODE system $\mathrm{d} {\mathbf {y}} = B \, {\mathbf {y}} \, \mathrm{d}t$. Calculating the inverse of $\varPhi (t)$ and considering long-time behaviour, Eq. (18) simplifies to

19 $$\begin{aligned} U(t) = - \int _0^t K(\tau ) \, V(t-\tau ) \, \mathrm{d}\tau + R(t), \end{aligned}$$

where memory kernel $K(\tau )$ is given by

20 $$\begin{aligned} K(\tau ) = \frac{\eta _1}{\lambda _1-\lambda _2} \left( \lambda _1 \exp (\lambda _2 \, \tau ) - \lambda _2 \exp (\lambda _1 \, \tau ) \right) \end{aligned}$$

and noise term *R*(*t*) is Gaussian with zero mean and the equilibrium correlation function satisfying the generalized fluctuation-dissipation theorem in the form

21 $$\begin{aligned} \langle R(t_1) R(t_2) \rangle = \frac{\eta _4^2}{2 \eta _1 \eta _2 \eta _3} \, K(t_2-t_1). \end{aligned}$$

Using (17), memory kernel (20) can be rewritten as

22 $$\begin{aligned} K(\tau ) = \eta _1 \, \exp \left( - \frac{\eta _2 \, \tau }{2} \right) \, \left( \cosh \left( \mu \, \tau \right) + \frac{\eta _2}{2 \mu } \sinh \left( \mu \, \tau \right) \right) , \end{aligned}$$

where $\mu = \sqrt{\eta _2^2/4 - \eta _3}$. We note that the auxiliary coefficient $\mu$ is a square root of a real negative number for $\eta _2^2 < 4 \eta _3$. However, formula (22) is still valid in this case: for $\eta _2^2 < 4 \eta _3$ it can be rewritten in terms of sine and cosine functions, taking into account that $\mu = {\mathrm {i}} \, |\mu |$ is pure imaginary, $\sinh ({\mathrm {i}} \, |\mu | \, \tau ) = {\mathrm {i}} \, \sin (|\mu |) \, \tau$ and $\cosh ({\mathrm {i}} \, |\mu | \, \tau ) = \cos (|\mu | \, \tau ).$

**Fig. 1:**
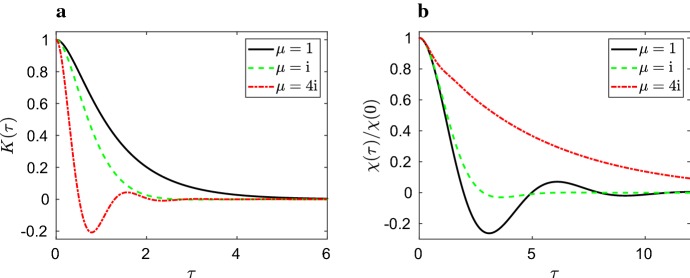
**a** Memory kernel $K(\tau )$ given by Eq. (22) for $\eta _1 = 1$, $\eta _2 = 4$ and three different values of $\eta _3$, namely $\eta _3 = 3$ (solid line, $\mu =1$), $\eta _3 = 5$ (dashed line, $\mu ={\mathrm {i}}$) and $\eta _3 = 20$ (dot-dashed line, $\mu =4 {\mathrm {i}}$). **b** Normalized velocity autocorrelation function $\chi (\tau )/\chi (0)$ computed by using Eq. (25) for the same parameter values as in panel (**a**)

The memory kernel $K(\tau )$, given by Eq. (22), is plotted in Fig. 1a for different values of parameter $\mu$. For simplicity, we use non-dimensionalized versions of our equations with dimensionless parameters $\eta _1 = 1$ and $\eta _2 = 4$. We choose three different values of $\eta _3$ so that the values of $\mu$ are 1, ${\mathrm {i}}$ and $4 {\mathrm {i}}$. In Fig. 1b, we plot the equilibrium velocity autocorrelation function which is defined as

$$\begin{aligned} \chi (\tau ) = \lim _{t \rightarrow \infty } \langle V(t) \, V(t-\tau ) \rangle , \end{aligned}$$

for $\tau \in [0,\infty )$. More precisely, we plot $\chi (\tau )/\chi (0)$ which is normalized so that its value at $\tau =0$ is equal to 1. It is related to the memory kernel by

23 $$\begin{aligned} \frac{\chi (\tau )}{\chi (0)} = \mathscr {L}^{-1} \, \left( \frac{1}{s + \mathscr {L}\big [K\big ](s)} \right) , \end{aligned}$$

where $\mathscr {L}\big [K\big ](s) = \int _0^\infty K(\tau ) \exp (-s \tau ) \, \mathrm{d}\tau$ is the Laplace transform of the memory kernel $K(\tau )$ and $\mathscr {L}^{-1}$ denotes Laplace inversion. Following Erban and Chapman (2019), we evaluate the right hand side of Eq. (23) as follows. Substituting Eq. (22) into (23), we obtain

24 $$\begin{aligned} \frac{\chi (\tau )}{\chi (0)} = \mathscr {L}^{-1} \left( \frac{s^2 + \eta _2 s + \eta _3}{s^3 + \eta _2 s^2 + (\eta _1 + \eta _3) s + \eta _1 \eta _2} \right) . \end{aligned}$$

The polynomial in the denominator, $p(s)=s^3 + \eta _2 s^2 + (\eta _1 + \eta _3) s + \eta _1 \eta _2,$ has positive coefficients. Since $p(-\eta _2)< 0 < p(0)$, it has one negative real root in interval $(-\eta _2,0)$, which we denote by $a_1$. The other two roots ($a_2$ and $a_3$ say) may be real or complex, but if they are complex they will be complex conjugates since *p*(*s*) has real coefficients. Assuming that the real part of each root is negative, we first find the partial fraction decomposition of the rational function in (24) as

$$\begin{aligned} \frac{s^2 + \eta _2 s + \eta _3}{s^3 + \eta _2 s^2 + (\eta _1 + \eta _3) s + \eta _1 \eta _2} = \frac{c_1}{s-a_1} + \frac{c_2}{s-a_2} + \frac{c_3}{s-a_3}, \end{aligned}$$

where $c_i \in {\mathbb {C}}$ are constants (which depend on $\eta _1$, $\eta _2$ and $\eta _3$). Then we can rewrite Eq. (23) as

25 $$\begin{aligned} \frac{\chi (\tau )}{\chi (0)} = c_1 \exp (a_1 \tau ) + c_2 \exp (a_2 \tau ) + c_3 \exp (a_3 \tau ). \end{aligned}$$

The results computed by (25) are shown in Fig. 1b. We note that although Eq. (25) may include complex exponentials, the resulting $\chi (\tau )$ is always real. Since the diffusion constant, *D*, and the second moment of the equilibrium velocity distribution, $\langle V^2 \rangle$, are related to $\chi$ by

$$\begin{aligned} D = \int _0^\infty \chi (\tau ) \, \mathrm{d}\tau = \frac{\eta _4^2}{2 \, \eta _1^2 \, \eta _2^2} \qquad \text{ and } \qquad \langle V^2 \rangle = \chi (0) = \frac{\eta _4^2}{2 \, \eta _1 \, \eta _2 \, \eta _3}, \end{aligned}$$

the parametrization (14) guarantees that both the value of $\chi (0)$ and the integral of $\chi (\tau )$ are captured accurately. However, the simplified SCG description (10)–(13) is not suitable to perfectly fit the velocity autocorrelation function or the memory kernel for all values of $\tau \in [0,\infty )$. In order to do this, we have to consider the SCG model (6)–(9) for larger values of *N* as it is done in the following section.

## General linear SCG model and autocorrelation functions

Considering the linear SCG model (6)–(9) for general values of *N*, we can solve Eqs. (8)–(9) for each value of $j=1$, 2, $\ldots$, *N* to generalize our previous result (19) as

26 $$\begin{aligned} U_{j,i}(t) = - \int _0^t K_j(\tau ) \, V_i(t-\tau ) \, \mathrm{d}\tau + R_{j,i}(t), \end{aligned}$$

where kernel $K_j(\tau )$ is given by [compare with (22)]

27 $$\begin{aligned} K_j(\tau ) = \eta _{j,1} \, \exp \left( - \frac{\eta _{j,2} \, \tau }{2} \right) \, \left( \cosh \left( \mu _j \, \tau \right) + \frac{\eta _{j,2}}{2 \mu _j} \sinh \left( \mu _j \, \tau \right) \right) \end{aligned}$$

with

28 $$\begin{aligned} \mu _j = \sqrt{\frac{\eta _{j,2}^2}{4} - \eta _{j,3}} \end{aligned}$$

and noise term $R_{j,i}(t)$ is Gaussian with zero mean and the equilibrium correlation function satisfying

$$\begin{aligned} \langle R_{j,i}(t_1) R_{j,i}(t_2) \rangle = \frac{\eta _{j,4}^2}{2 \, \eta _{j,1} \, \eta _{j,2} \, \eta _{j,3}} \, K_j(t_2-t_1). \end{aligned}$$

Substituting (26) to (7), we obtain the generalized Langevin equation

29 $$\begin{aligned} \frac{\mathrm{d}V_i}{\mathrm{d}t} = - \int _0^t K(\tau ) \, V_i(t-\tau ) \, \mathrm{d}\tau + R_i(t), \end{aligned}$$

where

30 $$\begin{aligned} K(\tau ) = \sum _{j=1}^N K_j(\tau ) \qquad \text{ and } \qquad R_i(t) = \sum _{j=1}^N R_{j,i}(t). \end{aligned}$$

In particular, we have 3*N* parameters to fit memory kernel $K(\tau )$, which can be estimated from all-atom MD simulations. There have been a number of approaches developed in the literature to estimate the memory kernel from MD simulations. Shin et al. (2010) use an integral equation with relates memory kernel $K(\tau )$ with the autocorrelation function for the force and the correlation function between the force and the velocity. Estimating these correlation functions from long time MD simulations and solving the integral equation, they obtain memory kernel $K(\tau )$. Other methods to estimate the memory kernel, $K(\tau )$, of the corresponding generalized Langevin equation (29) have been presented by Gottwald et al. (2015) and Jung et al. (2017).

An alternative approach to parametrize the linear SCG model (6)–(9) is to estimate the velocity autocorrelation function, $\chi (\tau )$, from all-atom MD simulations. This can be done by computing how correlated is the current velocity (at time *t*) with velocity at previous times. Since Eqs. (10)–(13) are linear SDEs, we can follow Mao (2007) to solve them analytically, using eigenvalues and eigenvectors of matrices appearing in their corresponding matrix formulation. Using this analytic solution, Erban (2016) use an acceptance-rejection algorithm to fit the parameters of linear SCG model (6)–(9) for $N=3$ to match the velocity autocorrelation functions of ions estimated from all-atom MD simulations of $\hbox {Na}^+$ and $\hbox {K}^+$ in the SPC/E water.

Since the parameter $\mu _j$ given by (28) is a square root of a real number, it can be both positive or purely imaginary. In particular, kernels $K_j(\tau )$ given by Eq. (27) can include both exponential, sine and cosine functions as illustrated in Fig. 1a. Since memory kernel $K(\tau )$ is given as the sum of $K_j(\tau )$ in Eq. (30), typical memory kernels and correlation functions estimated from all-atom MD simulations can be successfully matched by linear SCG models for relatively small values of *N*. However, as shown by Mao (2007), analytic solutions of linear SDEs also imply that the process is Gaussian at any time $t>0$, provided that we start with deterministic initial conditions. Thus the linear SCG model (6)–(9) for abtitrary values of *N* can only fit distributions which are Gaussian. This motivates our investigation of the nonlinear SCG model in the next two sections.

## Nonlinear SCG model for $N=1$

We begin by considering the nonlinear SCG model (2)–(5) for $$N=1$$. As in Sect. 2, we simplify our notation by dropping some subscripts and denoting $$X = X_i,$$$$V = V_i$$, $$U = U_{1,i}$$, $$Z=Z_{1,i}$$, $$W=W_{1,i}$$, $$g=g_j$$, $$h=h_j$$ and $$\eta _{k} = \eta _{1,k}$$ for $$k=1$$, 2, 3, 4. Then Eqs. (2)–(5) read as follows

31 $$\begin{aligned} \mathrm{d}X= & {} V \, \mathrm{d}t, \end{aligned}$$

32 $$\begin{aligned} \mathrm{d}V= & {} U \, \mathrm{d}t,\end{aligned}$$

33 $$\begin{aligned} \mathrm{d}U= & {} \left( -\eta _{1} V + h(Z) \right) \, g^\prime (g^{-1}(U)) \, \mathrm{d}t, \end{aligned}$$

34 $$\begin{aligned} \mathrm{d}Z= & {} - \left( \eta _{2} \, h(Z) + \eta _{3} \, U \right) \, \mathrm{d}t + \eta _{4} \; \mathrm{d}W, \qquad \end{aligned}$$

where *X* denotes (one coordinate of) the position of the coarse-grained particle, *V* is its velocity, *U* is its acceleration, *Z* is an auxiliary variable, $\mathrm{d}W$ is white noise, $\eta _j$, for $j=1$, 2, 3, 4, are positive parameters and functions $g: {\mathbb {R}} \rightarrow {\mathbb {R}}$ and $h: {\mathbb {R}} \rightarrow {\mathbb {R}}$ are yet to be specified.

Equation (31) describes the time evolution of the position, while Eqs. (32)–(34) admit a stationary distribution. We denote it by *p*(*v*, *u*, *z*). Then $p(v,u,z) \, \mathrm{d}v \, \mathrm{d}u \, \mathrm{d}z$ gives the probability that $V(t) \in [v,v+\mathrm{d}v)$, $U(t) \in [u,u+\mathrm{d}u)$ and $Z(t) \in [z,z+\mathrm{d}z)$ at equilibrium. The stationary distribution, *p*(*v*, *u*, *z*), of SDEs (32)–(34) can be obtained by solving the corresponding stationary Fokker-Planck equation

$$\begin{aligned} \frac{\eta _4^2}{2} \frac{\partial ^2 p}{\partial ^2 z} (v,u,z)= & {} \frac{\partial }{\partial v} \Big ( u \, p(v,u,z) \Big ) + \frac{\partial }{\partial u} \Big ( \big ( - \eta _1 v + h(z) \big ) g^\prime (g^{-1}(u)) \, p(v,u,z) \Big ) \\&+ \frac{\partial }{\partial z} \Big ( \big ( - \eta _2 h(z) - \eta _3 u \big ) p(v,u,z) \Big ), \end{aligned}$$

which gives

35 $$\begin{aligned} p(v,u,z) = \frac{C}{g^\prime (g^{-1}(u))} \, \exp \left[ - \frac{2 \eta _2}{\eta _4^2} \left( \eta _1 \eta _3 \, \frac{v^2}{2} + \eta _3 \, G \big ( g^{-1}(u) \big ) + H(z) \right) \right] , \end{aligned}$$

where *C* is the normalization constant, and functions *G* and *H* are integrals of functions *g* and *h*, respectively, which are given by

36 $$\begin{aligned} G(y) = \int _0^y g(\xi ) \, \mathrm{d}\xi \qquad \text{ and } \qquad H(y) = \int _0^y h(\xi ) \, \mathrm{d}\xi . \end{aligned}$$

We note that for the special case where *g* and *h* are given as identities, i.e. $g(y) = h(y)=y$ for $y \in {\mathbb {R}}$, the nonlinear SCG model (31)–(34) is equal to the linear SCG model (10)–(13) and functions *G* and *H* are $G(y)=H(y)=y^2/2$. Then the stationary distribution (35) is product of Gaussian distributions in *v*, *u* and *z* variables. In particular, we can easily calculate the second moments of these distributions in terms of parameters $\eta _j$. Estimating these moments from all-atom MD simulations, we can parametrize the resulting linear SCG model (10)–(13) as shown in Eq. (14). However, if we want to match a non-Gaussian force distribution, we have to consider nonlinear models. A simple one-parameter example is studied in the next section.

### One-parameter nonlinear function

Consider that *g* is a function depending on one additional positive parameter $\eta _5$ as follows

37 $$\begin{aligned} g(y) = |y|^{1/\eta _5} \mathrm{sign}y, \end{aligned}$$

where we use $\mathrm{sign}$ to denote the sign (signum) function

38 $$\begin{aligned} \mathrm{sign}y = \left\{ \begin{array}{rl} - 1, &{} \qquad \text{ for } \; y < 0, \\ 0, &{} \qquad \text{ for } \; y =0, \\ 1, &{} \qquad \text{ for } \; y > 0. \\ \end{array} \right. \end{aligned}$$

The function defined by (37) only satisfies our assumptions on *g* for $\eta _5 \in (0,1]$ as it is not differentiable at $y=0$ for $\eta _5>1$, but we will proceed with our analysis for any positive $\eta _5>0$. Consider that function *h* is an identity, i.e. $h(y)=y$ for $y \in {\mathbb {R}}$, then Eqs. (31)–(34) reduce to

39 $$\begin{aligned} \mathrm{d}X= & {} V \, \mathrm{d}t, \end{aligned}$$

40 $$\begin{aligned} \mathrm{d}V= & {} U \, \mathrm{d}t, \end{aligned}$$

41 $$\begin{aligned} \mathrm{d}U= & {} \left( -\eta _{1} V + Z \right) \, \eta _5^{-1} \, |U|^{1- \eta _5} \, \mathrm{d}t, \end{aligned}$$

42 $$\begin{aligned} \mathrm{d}Z= & {} - \left( \eta _{2} \, Z + \eta _{3} \, U \right) \, \mathrm{d}t + \eta _{4} \; \mathrm{d}W, \qquad \end{aligned}$$

where we would have to be careful, if we used this model to numerically simulate trajectories for $\eta _5>1$, because of possible division by zero for $U=0$ in Eq. (41). If $\eta _5 \in (0,1]$, then we do not have such technical issues. Using Eq. (35), the stationary distribution is equal to

43 $$\begin{aligned} p(v,u,z) = C |u|^{\eta _5-1} \, \exp \left[ - \frac{\eta _2}{\eta _4^2} \left( \eta _1 \eta _3 \, v^2 + \frac{2 \eta _3 \eta _5}{1+\eta _5} |u|^{1+\eta _5} + \, z^2 \right) \right] , \end{aligned}$$

where the normalization constant *C* is given by

$$\begin{aligned} \int _{-\infty }^\infty \int _{-\infty }^\infty \int _{-\infty }^\infty p(v,u,z) \, \mathrm{d}v\, \mathrm{d}u\, \mathrm{d}z= 1. \end{aligned}$$

Integrating (43), we get

$$\begin{aligned} C = \frac{\eta _2 \sqrt{\eta _1\eta _3}}{\pi \eta _4^2} \left( \frac{\eta _2 \eta _3 \eta _5}{\eta _4^2} \right) ^{\eta _5/(1+\eta _5)} \left( \frac{1+\eta _5}{2} \right) ^{1/(1+\eta _5)} \frac{1}{\Gamma \left( \frac{\eta _5}{1+\eta _5} \right) } \;, \end{aligned}$$

where ${\Gamma }$ is the gamma function defined as

44 $$\begin{aligned} {\Gamma }(s) = \int _0^\infty \xi ^{s-1} \exp (-\xi ) \, \mathrm{d} \xi . \end{aligned}$$

Let $\alpha \ge 0$. Integrating (43), we get

45 $$\begin{aligned} \langle |U|^\alpha \rangle = \left( \frac{\eta _4^2 \left( 1+\eta _5 \right) }{2 \eta _2 \eta _3 \eta _5} \right) ^{\alpha /(1+\eta _5)} \frac{\Gamma \left( \frac{\alpha +\eta _5}{1+\eta _5} \right) }{\Gamma \left( \frac{\eta _5}{1+\eta _5} \right) }. \end{aligned}$$

Using (45) for $\alpha =2$ and $\alpha =4$, we obtain the following expression for kurtosis

46 $$\begin{aligned} \mathrm {Kurt}[U] = \frac{\langle U^4 \rangle }{\langle U^2 \rangle ^2} = \Gamma \left( \frac{\eta _5}{1+\eta _5} \right) \Gamma \left( \frac{4+\eta _5}{1+\eta _5} \right) \left( \Gamma \left( \frac{2+\eta _5}{1+\eta _5} \right) \right) ^{-2}. \end{aligned}$$

**Fig. 2:**
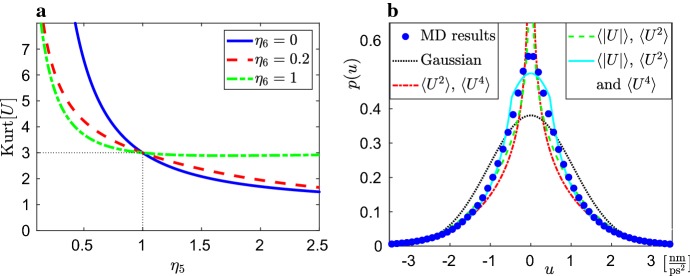
**a** Kurtosis $\mathrm {Kurt}[U]$ given by Eq. (59) as a function of parameter $\eta _5$ for three different values of parameter $\eta _6$. The result for $\eta _6=0$ (blue solid line) corresponds to the case of one-parameter function *g*, defined by (37), where the kurtosis is given by (46). **b** Distribution of *U* estimated from a long-time MD simulation (blue circles) compared with the results obtained by the linear SCG model (10)–(13) (black dotted line), nonlinear SCG models (31)–(34) with one-parameter function *g*, defined by (37), fitting $\langle U^2 \rangle$ and $\langle U^4 \rangle$ (red dot-dashed line) and $\langle |U| \rangle$ and $\langle U^2 \rangle$ (green dashed line), and the nonlinear SCG model (31)–(34) with two-parameter function *g* defined by (52), matching all three moments $\langle |U| \rangle$, $\langle U^2 \rangle$ and $\langle U^4 \rangle$ (cyan solid line) (color figure online)

In particular, the kurtosis is only a function of one parameter, $\eta _5$. It is plotted in Fig. 2a as the blue solid line, together with the kurtosis obtained for a more general two-parameter SCG model studied in Sect. 4.2. We observe that the distribution of *U* is leptokurtic for $\eta _5<1$ and platykurtic for $\eta _5>1$. If $\eta _5$ is equal to 1, then our SCG model given by Eqs. (31)–(34) reduces to the linear SCG model given by Eqs. (10)–(13), i.e. the stationary distribution is Gaussian and its kurtosis is 3. This is shown by the dotted line in Fig. 2a.

Since Eq. (46) only depends on parameter $\eta _5$, we can use the kurtosis of the acceleration distribution (which is equal to the kurtosis of the force distribution) esimated from MD simulations to find the value of parameter $\eta _5.$ To calculate the kurtosis, we estimate the fourth moment $\langle U^4 \rangle$ in addition to the second moment, $\langle U^2 \rangle$, used before in our estimating proceduce (14) for the linear model. In particular, we not only get Eq. (46) for calculating the value of parameter $\eta _5$, but also a restriction on other parameters $\eta _2$, $\eta _3$ and $\eta _4$. Using (45) for $\alpha =2$, it can be stated as follows

47 $$\begin{aligned} \frac{\eta _4^2}{2 \, \eta _2 \, \eta _3} = \frac{\eta _5}{1+\eta _5} \left( \frac{1+\eta _5}{\pi } \, \sin \left( \frac{\pi }{1+\eta _5} \right) \langle U^2 \rangle \right) ^{ (1+\eta _5)/2} \left( \Gamma \left( \frac{\eta _5}{1+\eta _5} \right) \right) ^{ 1+\eta _5} , \end{aligned}$$

where we have used properties of the gamma function, including $\Gamma (1+y)=y \, \Gamma (y)$ and Euler’s reflection formula, $\Gamma (1-y) \Gamma (y) \sin (\pi y) = \pi$, to simplify the right hand side. We note that in the Gaussian case, $\eta _5=1$, the right hand side of Eq. (47) further simplifies to

48 $$\begin{aligned} \frac{\eta _4^2}{2 \, \eta _2 \, \eta _3} = \langle U^2 \rangle , \end{aligned}$$

which is indeed the formula for the second moment of *U* given by the linear SCG model (10)–(13). Equation (47) provides one restriction on four remaining parameters, $$\eta _1$$, $$\eta _2$$$$\eta _3$$ and $$\eta _4$$, which need to be specified. This can be done by estimating three additional statistics from MD simulations, as in the case of the linear SCG model (10)–(13) in Eq. (14). Indeed, the stationary distributions of *V* and *Z* are Gaussian with mean zero. Their second moments and the diffusion constant, *D*, for the nonlinear SCG model (31)–(34) can be calculuted as

49 $$\begin{aligned} D = \frac{\eta _4^2}{2 \, \eta _1^2 \, \eta _2^2}, \qquad \langle V^2 \rangle = \frac{\eta _4^2}{2 \, \eta _1 \, \eta _2 \, \eta _3} \qquad \text{ and } \qquad \langle Z^2 \rangle = \frac{\eta _4^2}{2 \, \eta _2}. \end{aligned}$$

Therefore, assuming that *D*, $\langle V^2 \rangle$, $\langle Z^2 \rangle$ are obtained from MD simulations and $\eta _4^2/(2 \eta _2 \eta _3)$ is given by (47), we can calculate parameters $\eta _k$ by

50 $$\begin{aligned} \displaystyle \eta _1= & {} \displaystyle \frac{1}{\langle V^2 \rangle } \left( \frac{\eta _4^2}{2 \, \eta _2 \, \eta _3} \right) , \qquad \eta _2 = \displaystyle \frac{\langle Z^2 \rangle \, \langle V^2 \rangle ^2}{D} \left( \frac{\eta _4^2}{2 \, \eta _2 \, \eta _3} \right) ^{ -2}, \end{aligned}$$

51 $$\begin{aligned} \displaystyle \eta _3= & {} \displaystyle \langle Z^2 \rangle \left( \frac{\eta _4^2}{2 \, \eta _2 \, \eta _3} \right) ^{ -1}, \qquad \eta _4 = \displaystyle \sqrt{\frac{2}{D}} \, \langle Z^2 \rangle \, \langle V^2 \rangle \left( \frac{\eta _4^2}{2 \, \eta _2 \, \eta _3} \right) ^{ -1}. \end{aligned}$$

We note that in the Gaussian case, $\eta _5=1$, we can substitute Eq. (48) for $\eta _4^2/(2 \eta _2 \eta _3)$ and the parametrization approach (50)–(51) simplifies to Eq. (14) used in the case of the linear SCG model (10)–(13). In the next subsection, we generalize formula (37) to a two-parameter function and show that the parametrization approach (50)–(51) is still applicable to the case of more general SCG models.

### Two-parameter nonlinear function

Consider that *g* is a function depending on two positive parameters $\eta _5$ and $\eta _6$ as follows

52 $$\begin{aligned} g(y) = \left\{ \begin{array}{ll} 0, &{} \quad \displaystyle \text{ for } \; |y| \le \eta _6^{\eta _5} (1-\eta _5), \\ \displaystyle \left( \eta _6 \left( 1 - \frac{1}{\eta _5}\right) + \frac{\eta _6^{1-\eta _5}}{\eta _5} |y| \right) \mathrm{sign}y, &{} \quad \displaystyle \text{ for } \; \eta _6^{\eta _5} (1-\eta _5) < |y| \le \eta _6^{\eta _5}, \\ \displaystyle \left| y \right| ^{1/\eta _5} \mathrm{sign}y, &{} \quad \displaystyle \text{ for } \; |y| > \eta _6^{\eta _5}, \\ \end{array} \right. \end{aligned}$$

where $\mathrm{sign}$ function is defined by (38). In particular, our expression for function *g* is equal to the formula (37) for sufficiently large values of |*y*|. As discussed in the previous section, if we used formula (37), there would be some issues for *y* close to zero [for example, the division by zero for $U=0$ and $\eta _5>1$ in Eq. (41)], so our generalized formula (52) replaces (37) with a linear function for smaller values of |*y*|. On the face of it, it looks that there could also be some issues with the generalized formula (52), because it is not strictly increasing for $|y| \le \eta _6^{\eta _5} (1-\eta _5)$. However, function (52) is increasing and invertible away of this region with its inverse given by

$$\begin{aligned} g^{-1}(u) = \left\{ \begin{array}{ll} \displaystyle \eta _5 \eta _6^{\eta _5-1} \left( |u| - \eta _6 \left( 1 - \frac{1}{\eta _5}\right) \right) \mathrm{sign}\, u, &{} \quad \displaystyle \text{ for } \; 0 < |u| \le \eta _6, \\ \displaystyle \left| u \right| ^{\eta _5} \mathrm{sign}\,u, &{} \quad \displaystyle \text{ for } \; |u| > \eta _6. \\ \end{array} \right. \end{aligned}$$

Moreover, what we really need in Eqs. (31)–(34) is $g^\prime (g^{-1}(u))$ which can be defined as the following continuous function

53 $$\begin{aligned} g^\prime (g^{-1}(u)) = \frac{1}{\eta _5} \times \left\{ \begin{array}{ll} \displaystyle \eta _6^{1-\eta _5}, &{} \quad \displaystyle \text{ for } \; |u| \le \eta _6, \\ \displaystyle \left| u \right| ^{1-\eta _5}, &{} \quad \displaystyle \text{ for } \; |u| > \eta _6, \\ \end{array} \right. \end{aligned}$$

where the removable discontinuity at $u=0$ has disappeared because we have defined $g^\prime (g^{-1}(0))=\eta _6^{1-\eta _5}/\eta _5$. Integrating (52) and substituting (53), we get

54 $$\begin{aligned} G\big (g^{-1}(u)\big ) = \left\{ \begin{array}{ll} \displaystyle \frac{\eta _5\eta _6^{\eta _5-1}}{2} u^2, &{} \quad \displaystyle \text{ for } \; |u| \le \eta _6, \\ \displaystyle \frac{\eta _5(\eta _5-1) \eta _6^{1+\eta _5}}{2 (1+\eta _5)} + \frac{\eta _5}{1+\eta _5} |u|^{1+\eta _5}, &{} \quad \displaystyle \text{ for } \; |u| > \eta _6, \\ \end{array} \right. \end{aligned}$$

where *G* is the integral of function *g* defined by (36). Consider again that *h* is an identity, i.e. $h(y)=y$ for $y \in {\mathbb {R}}$. Then the stationary distribution (35) is again Gaussian in *V* and *Z* variables with their second moments given by Eq. (49). Let us denote the marginal stationary distribution of *U* by

$$\begin{aligned} p_u(u) = \int _{-\infty }^\infty \int _{-\infty }^\infty p(v,u,z) \, \mathrm{d}v\, \mathrm{d}z. \end{aligned}$$

Using (35) and (54), we have

55 $$\begin{aligned} p_u(u) = \left\{ \begin{array}{ll} \displaystyle C_u \, \eta _6^{\eta _5-1} \, \exp \left[ - \frac{\eta _2 \eta _3 \eta _5 \eta _6^{1+\eta _5}}{\eta _4^2} \left( \frac{u^2}{\eta _6^{2}} + \frac{1-\eta _5}{1+\eta _5} \right) \right] , &{} \quad \text{ for } \; |u| \le \eta _6, \\ \displaystyle C_u \, |u|^{\eta _5-1} \, \exp \left[ - \frac{2 \eta _2 \eta _3 \eta _5}{\eta _4^2 (1+\eta _5)} |u|^{1+\eta _5} \right] , &{} \quad \text{ for } \; |u| > \eta _6, \\ \end{array} \right. \end{aligned}$$

where $C_u$ is the normalization constant given by

$$\begin{aligned} \int _{-\infty }^\infty p_u(u) \, \mathrm{d}u= 1. \end{aligned}$$

Let us define

56 $$\begin{aligned} \kappa _1 = \frac{\eta _2 \eta _3 \eta _5 \eta _6^{1+\eta _5}}{\eta _4^2} \qquad \text{ and } \qquad \kappa _2 = \frac{1}{1+\eta _5}. \end{aligned}$$

Integrating (55), we get, for any $\alpha \ge 0$,

57 $$\begin{aligned} \frac{\langle |U|^\alpha \rangle }{\eta _6^\alpha } = \frac{F(\kappa _1,\kappa _2,\alpha )}{F(\kappa _1,\kappa _2,0)}, \end{aligned}$$

where function $F(\kappa _1,\kappa _2,\alpha )$ is defined by

58 $$\begin{aligned} F(\kappa _1,\kappa _2,\alpha )= & {} \left( 2 \kappa _1 \kappa _2 \right) ^{(1-\alpha ) \kappa _2} \exp \left( 2 \kappa _1 \kappa _2 \right) \, \Gamma \big ( 1+(\alpha -1) \kappa _2, 2 \kappa _1 \kappa _2 \big ) \nonumber \\&+\, \kappa _1^{(1-\alpha )/2} \exp (\kappa _1) \, \gamma \bigg ( \frac{\alpha +1}{2}, \kappa _1 \bigg ) \end{aligned}$$

and $\Gamma$ (resp. $\gamma$) is the upper (resp. lower) incomplete gamma function defined by

$$\begin{aligned} \Gamma (s,y) = \int _y^\infty \xi ^{s-1} \exp (-\xi ) \, \mathrm{d} \xi , \qquad \gamma (s,y) = \int _0^y \xi ^{s-1} \exp (-\xi ) \, \mathrm{d} \xi . \end{aligned}$$

Substituting $\alpha =2$ and $\alpha =4$ in Eq. (57), we get

59 $$\begin{aligned} \mathrm {Kurt}[U] = \frac{\langle U^4 \rangle }{\langle U^2 \rangle ^2} = \frac{F(\kappa _1,\kappa _2,4) \, F(\kappa _1,\kappa _2,0)}{ (F(\kappa _1,\kappa _2,2))^2}. \end{aligned}$$

This formula for the kurtosis is visualized in Fig. 2a as a function of parameter $\eta _5$ for three different values of parameter $\eta _6$. We note that the case $\eta _6=0$ corresponds to the case studied in Sect. 4.1. If $\eta _6=0$, then Eq. (56) implies $\kappa _1=0$. Since $\gamma (s,0)=0$ and $\Gamma (s,0)=\Gamma (s)$, where $\Gamma (s)$ is the standard gamma function given by (44), we can confirm that Eq. (59) converges to our previous result (46) as $\eta _6 \rightarrow 0.$

Substituting $\alpha =1$ into (58), we obtain $F(\kappa _1,\kappa _2,1) = \exp \left( \kappa _1 \right) .$ Consequently, using $\alpha =1$ in Eq. (57), we obtain

60 $$\begin{aligned} \frac{\langle |U| \rangle }{\eta _6} = \frac{\exp \left( \kappa _1\right) }{F(\kappa _1,\kappa _2,0)}. \end{aligned}$$

Using $\alpha =2$ in Eq. (57), we get

61 $$\begin{aligned} \frac{\langle U^2 \rangle }{\langle |U| \rangle ^2} = \frac{F(\kappa _1,\kappa _2,2) \, F(\kappa _1,\kappa _2,0)}{\exp (2\kappa _1)}. \end{aligned}$$

Consequently, if we use MD simulations to estimate not only the second and fourth moments, $\langle U^2 \rangle$ and $\langle U^4 \rangle$, but also the first absolute moment $\langle |U| \rangle$, we can substitute the estimated MD values into Eqs. (59) and (61) to obtain two equations for two unknowns $\kappa _1$ and $\kappa _2$. Solving these two equations numerically, we can get $\kappa _1$ and $\kappa _2$. Then we can use (56) and (60) to get the original parameters $\eta _5$ and $\eta _6$ by

62 $$\begin{aligned} \eta _5 = \frac{1-\kappa _2}{\kappa _2} \qquad \text{ and } \qquad \eta _6 = \frac{\langle |U| \rangle \, F(\kappa _1,\kappa _2,0)}{\exp \left( \kappa _1\right) }. \end{aligned}$$

Moreover, Eq. (56) also implies the following restriction on other parameters $\eta _2$, $\eta _3$ and $\eta _4$

63 $$\begin{aligned} \frac{\eta _4^2}{\eta _2 \eta _3} = \frac{1-\kappa _2}{\kappa _1 \, \kappa _2 \, \exp \left( \kappa _1/\kappa _2 \right) } \Big ( \langle |U| \rangle \, F(\kappa _1,\kappa _2,0) \Big )^{1/\kappa _2}. \end{aligned}$$

This restriction is equivalent to restriction (47). Therefore, assuming again that *D*, $\langle V^2 \rangle$, $\langle Z^2 \rangle$ are obtained from MD simulations and $\eta _4^2/(2 \eta _2 \eta _3)$ is given by (63), we can calculate parameters $\eta _1$, $\eta _2$, $\eta _3$ and $\eta _4$ by Eqs. (50)–(51).

We note that the two additional parameters $\eta _5$ and $\eta _6$ can be used to satisfy both Eqs. (59) and (61), while in Sect. 4.1 we could only use one equation (Eq. (46) for kurtosis) to fit one parameter $\eta _5$. However, in the case of one-parameter function (37), we could (instead of fitting the kurtosis) match the quantity $\langle U^2 \rangle /\langle |U| \rangle ^2$ with MD simulations, i.e. we could replace Eq. (46) by Eq. (61) simplified to the one-parameter case corresponding to function (37). Passing to the limit $\eta _6 \rightarrow 0$ in Eq. (61) and using Euler’s reflection formula, $\Gamma (1-y) \Gamma (y) \sin (\pi y) = \pi$, we obtain that the one-parameter nonlinearity (37) implies the following formula

64 $$\begin{aligned} \frac{\langle U^2 \rangle }{\langle |U| \rangle ^2} = \frac{\pi }{1+\eta _5} \left( \sin \left( \frac{\pi }{1+\eta _5} \right) \right) ^{ -1}. \end{aligned}$$

Thus, in Sect. 4.1, we could use $\langle |U| \rangle$ and $\langle U^2 \rangle$ estimated from long-time MD simulations to calculate the left hand side of Eq. (64), which could then be used to select parameter $\eta _5$. Other parameters could again be chosen by Eqs. (50)–(51).

### Application to MD simulations

In Sects. 4.1 and 4.2, we have presented three approaches to fit nonlinear SCG models which have non-Gaussian force distributions to data obtained from MD simulations. In this section, we apply them to the results obtained by an illustrative MD simulation of a Lennard-Jones fluid, where we consider a box of 512 atoms which interact with each other through the Lennard-Jones force terms for parameters given for liquid argon (Rahman 1964), i.e. particles interact in pairs according to the Lennard-Jones potential $4 \varepsilon \, ( (\sigma /r)^{12} - (\sigma /r)^6 )$, where $\varepsilon /k_B = 120\,\hbox {K}$, $\sigma = 0.34\,\hbox {nm}$ and *r* being the distance between particles. We use standard NVT simulations where the temperature ($T=94.4\,\hbox {K}$) is controlled using the thermostat of Nosé (1984) and Hoover (1985) and the number of particles ($N=512$ in a cubic box of side $2.91\,\hbox {nm}$) is kept constant by implementing periodic boundary conditions.

Using a long time MD simulation (time series of lentgh $10\,\hbox {ns}$), we estimate three moments $\langle |U| \rangle$, $\langle U^2 \rangle$ and $\langle U^4 \rangle$ as averages over all three coordinates, i.e.

$$\begin{aligned} \langle |U|^\alpha \rangle = \frac{\langle |U_1|^\alpha \rangle + \langle |U_2|^\alpha \rangle + \langle |U_3|^\alpha \rangle }{3}, \qquad \quad \text{ for } \quad \alpha =1, 2 \; \text{ and } \; 4, \end{aligned}$$

where $(U_{1},U_{2},U_{3})$ is the acceleration of one specific atom (tagged particle) to which our SCG model is applied. Rounding all computational results to three significant figures, we obtain $\langle |U| \rangle = 0.753\,\hbox {nm}\,\hbox {ps}^{-2}$, $\langle U^2 \rangle = 1.10\,\hbox {nm}^2\,\hbox {ps}^{-4}$ and $\langle U^4 \rangle = 7.03\,\hbox {nm}^4\,\hbox {ps}^{-8}$.

In Fig. 2b, we plot the equilibrium MD distribution of the acceleration (average over all three coordinates) using blue circles. The resulting distribution is leptokurtic (with positive excess kurtosis). Its kurtosis has been estimated as $\mathrm {Kurt}[U]=5.85$. The numerical values on the *u*-axis in Fig. 2b are expressed in [nm ps$^{-2}$]. Since the acceleration, *U*, is proportional to the force exerted on the tagged particle (with the scaling factor equal to the atomic mass of argon), the plot of the acceleration distribution in Fig. 2b can also be interpreted as the plot of the force distribution, which has the same kurtosis, provided that we suitably rescale the units on the *u*-axis.

If we only attempt to fit the value of $\langle U^2 \rangle$, we could parametrize the linear SCG model (10)–(13), which leads to the Gaussian acceleration distribution (plotted as the black dotted line in Fig. 2b). Using the one-parameter nonlinear function (37) from Sect. 4.1, we can use Eq. (46) to find parameter $\eta _5=0.550$ so that the nonlinear SCG model gives the same kurtosis as observed in MD simulations ($\mathrm {Kurt}[U]=5.85$). The resulting distribution is given as the red dot-dashed line in Fig. 2b. It matches both second and fourth moments, $\langle U^2 \rangle$ and $\langle U^4 \rangle$.

Using all-atom MD simulations, we can not only estimate the kurtosis, but other dimensionless ratios of moments of *U*. For example, we obtain $\langle U^2 \rangle /\langle |U| \rangle ^2=1.93$. This estimate can be substituted in Eq. (64), which provides an alternative approach to obtain the value of parameter $\eta _5$ of the one-parameter nonlinear function (37). Using $\langle U^2 \rangle /\langle |U| \rangle ^2=1.93$ and solving Eq. (64) numerically, we obtain $\eta _5=0.692.$ The resulting distribution, which matches $\langle |U| \rangle$ and $\langle U^2 \rangle$, is plotted as the green dashed line in Fig. 2b. We note that the parameter $\eta _5$ is dimensionless, because both Eqs. (46) and (64) only depend on dimensionless quantities estimated from MD simulations. Since both distributions (for $\eta _5=0.550$ and $\eta _5=0.692$) are given by (43), they are unbounded for *u* close to zero. This motivates the choice of our two-parameter nonlinear function *g* used in Sect. 4.2.

Substituting $\mathrm {Kurt}[U]=5.85$ and $\langle U^2 \rangle /\langle |U| \rangle ^2=1.93$ in Eqs. (59) and (61) and solving them numerically, we obtain $\kappa _1=0.149$ and $\kappa _2=0.771$. Substituting into (62), we get the two parameters of model from Sect. 4.2 as $\eta _5=0.297$ and $\eta _6=0.472~\hbox {nm}\,\hbox {ps}^{-2}$. The resulting distribution, given by Eq. (55), is plotted in Fig. 2b as the cyan solid line. We observe that the distribution is now bounded. It is a piecewise defined function which is Gaussian for the values of *u* satisfying $|u| \le \eta _6$, which removes the singularity at $u=0$. At the same time, the distribution given by Eq. (55) matches all three moments estimated from MD simulations, $\langle |U| \rangle$, $\langle U^2 \rangle$ and $\langle U^4 \rangle$. As we can see in Fig. 2b, this distribution does not perfectly fit the acceleration distribution estimated from MD simulations. If our aim is to obtain a SCG model which better fits the whole distribution, we can use SCG models for larger values of *N* as we will discuss in the next section.

## Nolinear SCG model for general values of *N*

We have already observed in Sects. 2 and 3 that the linear SCG model (6)–(9) can match the MD values of a few moments for $N=1$, while we need to consider larger values of *N* to match the entire velocity autocorrelation function. Considering the nonlinear SCG model (2)–(5), we have two options to capture more details of the non-Gaussian force distribution observed in MD simulations. We could either keep $N=1$, as in Sect. 4, and introduce additional parameters into nonlinearity $g=g_1$, or we could consider larger values of *N*. In Sect. 4, we have shown that by going from one-parameter to two-parameter function *g*, we improve the match with MD results. In this section, we will discuss the second option: we will use larger values of *N*.

Consider equations corresponding to the *i*-coordinate, $i=1$, 2, 3, of the nonlinear SCG model (2)–(5). Let us denote the stationary distribution of Eqs. (3)–(5) by

$$\begin{aligned} p(v,{\mathbf {u}},{\mathbf {z}}) \equiv p(v,u_1,u_2,\ldots ,u_N,z_1,z_2,\ldots ,z_N). \end{aligned}$$

Then $p(v,{\mathbf {u}},{\mathbf {z}}) \, \mathrm{d}v \, \mathrm{d}u_1 \, \mathrm{d}u_2 \, \ldots \, \mathrm{d}u_N \, \mathrm{d}z_1 \, \mathrm{d}z_2 \, \ldots \, \mathrm{d}z_N$ gives the probability that $V_i(t) \in [v,v+\mathrm{d}v)$, $U_{j,i}(t) \in [u_j,u_j+\mathrm{d}u_j)$ and $Z_{j,i}(t) \in [z_j,z_j+\mathrm{d}z_j)$, for $j=1$, 2, $\ldots$, *N*, at equilibrium. The stationary distribution can be obtained by solving the corresponding stationary Fokker-Planck equation

65 $$\begin{aligned} \frac{\eta _{j,4}^2}{2} \frac{\partial ^2 p}{\partial ^2 z_j} (v,{\mathbf {u}},{\mathbf {z}})= & {} \frac{\partial }{\partial v} \left( p(v,{\mathbf {u}},{\mathbf {z}}) \, \sum _{j=1}^{N} u_j \right) \nonumber \\&+ \sum _{j=1}^{N} \frac{\partial }{\partial u_j} \Big ( \big ( - \eta _{j,1} v + h_j(z_j) \big ) g_j^\prime (g_j^{-1}(u_j)) \, p(v,{\mathbf {u}},{\mathbf {z}}) \Big ) \nonumber \\&+ \sum _{j=1}^{N} \frac{\partial }{\partial z_j} \Big ( \big ( - \eta _{j,2} h_j(z_j) - \eta _{j,3} u_j \big ) p(v,{\mathbf {u}},{\mathbf {z}}) \Big ). \end{aligned}$$

Our analysis in Sect. 4.1 shows that parameters $\eta _{j,2}$, $\eta _{j,3}$ and $\eta _{j,4}$ appear on the left hand side of Eq. (47) as a suitable fraction, which in the Gaussian case corresponds to the second moment of the acceleration (see Eq. (48)). Considering general *N*, we define this fraction as new parameters

$$\begin{aligned} \sigma _j = \frac{\eta _{j,4}^2}{2 \, \eta _{j,2} \, \eta _{j,3}}, \qquad \quad \text{ for } \qquad j=1,2,\ldots ,N, \end{aligned}$$

and we again assume that the second moment of the velocity distribution, $\langle V^2 \rangle = \langle V_i^2 \rangle$, can be estimated from long-time MD simulations. In order to find the stationary distribution, we will require that parameters $\eta _{j,1}$, $\eta _{j,2}$, $\eta _{j,3}$ and $\eta _{j,4}$ satisfy (compare with Eq. (49) for $N=1$)

$$\begin{aligned} \langle V^2 \rangle = \frac{\eta _{j,4}^2}{2 \, \eta _{j,1} \, \eta _{j,2} \, \eta _{j,3}} = \frac{\sigma _j}{\eta _{j,1}} \,, \qquad \quad \text{ for } \text{ all } \qquad j=1,2,\ldots ,N. \end{aligned}$$

Then the stationary distribution, obtained by solving (65), is given by

66 $$\begin{aligned} p(v,{\mathbf {u}},{\mathbf {z}}) = C \, \left( \prod _{j=1}^N \frac{1}{g_j^\prime (g_j^{-1}(u_j))} \right) \, \exp&\Bigg [ - \, \frac{v^2}{2 \, \langle V^2 \rangle } - \sum _{j=1}^N \frac{1}{\sigma _j} \, G_j \big ( g_j^{-1}(u_j) \big ) \nonumber \\&\quad - \sum _{j=1}^N \frac{2 \eta _{j,2}}{\eta _{j,4}^2} H_j(z_j) \Bigg ], \end{aligned}$$

where *C* is the normalization constant and functions $G_j$ and $H_j$ are integrals of functions $g_j$ and $h_j$, respectively, which are given by

$$\begin{aligned} G_j(y) = \int _0^y g_j(\xi ) \, \mathrm{d}\xi , \qquad H_j(y) = \int _0^y h_j(\xi ) \, \mathrm{d}\xi \,, \qquad \text{ for } \quad j=1,2,\ldots ,N. \end{aligned}$$

Following (37), we assume that $$h_j(z_j)=z_j$$ and each $$g_j$$ is a function of one additional positive parameter $$\eta _{j,5},$$$$j=1$$, 2, $$\ldots $$, *N*, given as

67 $$\begin{aligned} g_j(y) = \left| y \right| ^{1/\eta _{j,5}} \mathrm{sign}y. \end{aligned}$$

Then we have

$$\begin{aligned} g_j^\prime (g_j^{-1}(u_j)) = \frac{ \left| u_j \right| ^{1-\eta _{j,5}}}{\eta _{j,5}} \qquad \text{ and } \qquad G_j \big ( g_j^{-1}(u_j) \big ) = \frac{\eta _{j,5}}{1+\eta _{j,5}} |u_j|^{1+\eta _{j,5}}. \end{aligned}$$

Then the stationary distribution (66) is Gaussian in $V_i$ and $Z_{j,i}$ variables and we can integrate (66) to calculate the marginal distribution of $U_{j,i}$ by

$$\begin{aligned} p_j(u_j) = \int _{-\infty }^\infty \cdots \int _{-\infty }^\infty p(v,{\mathbf {u}},{\mathbf {z}}) \, \mathrm{d}v\, \mathrm{d}u_1 \, \mathrm{d}u_2 \, \ldots \, \mathrm{d}u_{j-1} \, \mathrm{d}u_{j+1} \, \ldots \, \mathrm{d}u_N \, \mathrm{d} {\mathbf {z}} \,. \end{aligned}$$

Consequently,

68 $$\begin{aligned} p_j(u_j) = C_j |u_j|^{\eta _{j,5}-1} \, \exp \left[ - \frac{\eta _{j,5}}{\sigma _j (1+\eta _{j,5})} |u_j|^{1+\eta _{j,5}} \right] , \end{aligned}$$

where the normalization constant $C_j$ is given by

$$\begin{aligned} \int _{-\infty }^\infty p_j(u_j) \, \mathrm{d}u_j = 1. \end{aligned}$$

Integrating (68), we can calculate

$$\begin{aligned} \langle |U_{j,i}|^\alpha \rangle = \int _{-\infty }^\infty |u_j|^\alpha p_j(u_j) \, \mathrm{d}u_j, \qquad \text{ for } \text{ any } \; \alpha \ge 0, \end{aligned}$$

as

69 $$\begin{aligned} \langle |U_{j,i}|^\alpha \rangle = \left( \frac{\sigma _j(1+\eta _{j,5})}{\eta _{j,5}} \right) ^{\alpha /(1+\eta _{j,5})} \frac{ \Gamma \left( \frac{\alpha +\eta _{j,5}}{1+\eta _{j,5}} \right) }{ \Gamma \left( \frac{\eta _{j,5}}{1+\eta _{j,5}} \right) }. \end{aligned}$$

The acceleration of the coarse-grained particle is given by

$$\begin{aligned} U_{i} = \sum _{j=1}^N U_{j,i}. \end{aligned}$$

Using the symmetry of (68), odd moments of $U_{j,i}$ are equal to zero. In particular, $\langle U_{j,i} \rangle = 0$ and $\langle U_{j,i}^3 \rangle = 0$ for $j=1$, 2, $\ldots$, *N*. Consequently,

70 $$\begin{aligned} \langle U_{i}^2 \rangle= & {} \sum _{j=1}^N \langle U_{j,i}^2 \rangle , \end{aligned}$$

71 $$\begin{aligned} \langle U_{i}^4 \rangle= & {} 3 \langle U_{i}^2 \rangle ^2 + \sum _{j=1}^N \langle U_{j,i}^4 \rangle - 3 \langle U_{j,i}^2 \rangle ^2, \end{aligned}$$

which gives

72 $$\begin{aligned} \mathrm {Kurt}[U_i] = \frac{\langle U_i^4 \rangle }{\langle U_i^2 \rangle ^2} = 3 + \frac{ \sum _{j=1}^N \langle U_{j,i}^4 \rangle - 3 \langle U_{j,i}^2 \rangle ^2 }{ \sum _{j=1}^N \langle U_{j,i}^2 \rangle }. \end{aligned}$$

Substituting Eq. (69) for moments on the right hand side of Eq. (72), we can express the kurtosis of $U_i$ in terms of 2*N* parameters $\sigma _j$ and $\eta _{j,5}$, where $j=1$, 2, $\ldots$, *N*. For example, if we choose the values of dimensionless parameters $\eta _{j,5}$ equal to given numbers and define new parameters

$$\begin{aligned} \kappa _j = \big ( \sigma _j \big )^{2/(1+\eta _{j,5})}, \end{aligned}$$

then Eq. (69) implies that $\langle U_{j,i}^2 \rangle$ is a linear function of $\kappa _j$ and $\langle U_{j,i}^4 \rangle$ is a quadratic function of $\kappa _j$. Equations (70) and (71) can then be rewritten as the following system of two equations for $\kappa _1, \kappa _2, \ldots , \kappa _N$

$$\begin{aligned} \sum _{i=1}^N c_{1,j} \kappa _j = \langle U_{i}^2 \rangle , \qquad \sum _{i=1}^N c_{2,j} \kappa _j^2 = \langle U_{i}^4 \rangle - 3 \langle U_{i}^2 \rangle ^2, \end{aligned}$$

where $c_{1,j}$ and $c_{2,j}$ are known constants, which will depend on our initial choice of values of $\eta _{j,5}$. Thus, using $N>2$, we still have an opportunity to not only fit the second and fourth moments of the force distribution, but other moments as well. For example, the 6-th moment, $\langle U_{i}^6 \rangle$, would include the linear combination of the third powers of $\kappa _j$. We could also fit other properties of the force distribution estimated from MD simulations. For example, we could generalize one-parameter nonlinearities (67) to two-parameter nonlinear functions, as we did in Eq. (52). Then we could match the value of the distribution at $u=0$, if our aim was to get a better fit of the MD acceleration distribution obtained in the illustrative example in Fig. 2b. Another possible generalization is to consider nonlinear functions $h_j$, provided that we estimate more statistics on the auxiliary variable *Z* from MD simulations.

## Discussion and conclusions

We have presented and analyzed a family of SCG models given by Eqs. (2)–(5), which can be parametrized to fit properties of detailed all-atom MD models. A special choice of functions $g_j$ and $h_j$ in Eqs. (2)–(5) leads to the linear SCG model (6)–(9) which is used in a multiscale (multi-resolution) method developed by Erban (2016) as an intermediate description between all-atom MD simulations and BD models. The linear SCG model is studied in more detail in Sects. 2 and 3, where we highlight that 4*N* parameters of this model can match some statistics estimated from all-atom MD simulations with increased accuracy as we increase *N*, but there are also statistics which cannot be matched for any value of *N*. They include non-Gaussian force distributions.

In Sects. 2 and 3, we show that the linear SCG model (6)–(9) corresponds to the generalized Langevin equation with the stochastic driving force being Gaussian. Such systems have been analysed since the work of Kubo (1966). One approach to match non-Gaussian MD force distributions could be to use the non-Gaussian generalized Langevin equation which was analyzed by Fox (1977) using methods of multiplicative stochastic processes. However, if we want to generalize the linear SCG model (6)–(9) while keeping its structure as a relatively low-dimensional system of SDEs, then it can be done by introducing nonlinear functions $g_j$ and $h_j$ as shown in Eqs. (2)–(5). The advantage of the presented approach is that we can directly replace the linear model by Eqs. (2)–(5) in multiscale methods which use all-atom MD simulations in parts of the computational domain and (less detailed) BD simulations in the remainder of the domain. Coupling MD and BD models is a possible approach to incorporate atomic-level information into models of intracellular processses which include transport of molecules between different parts of the cell (Erban 2014, 2016; Gunaratne et al. 2019).

The nonlinear SCG model (2)–(5) is studied in Sect. 4 for $N=1$. Describing the nonlinearity as the one-parameter function given by (37), we can use its dimensionless parameter $\eta _5$ to match the kurtosis of the force distribution estimated from all-atom MD simulations. Although the one-parameter case is easy to analyze in terms of the gamma function, it has some undesirable properties for small forces. If $\eta _5>1$, we can obtain large terms in the dynamical equation (41) for small values of *U*; this corresponds to the zero value of stationary probability distribution (43) for $u=0$. If $\eta _5<1$, we have small terms in the dynamical equation (41), but the stationary probability distribution (43) is unbounded for $u=0$. In Sect. 4.2, we have shown that these issues can be avoided if the two-parameter nonlinear function (52) is used instead of the one-parameter function (37). The resulting equations are solved in terms of incomplete gamma functions. In Sect. 5, we study the nonlinear model for general values of *N* where each $g_j$ is a one-parameter nonlinearity given by Eq. (67). However, we could also consider two-parameter functions $g_j$, like we did in Eq. (52) for $N=1$, to improve the properties of the SCG model for general values of *N*.
